# Burden of anemia and its association with HAART in HIV infected children in Ethiopia: a systematic review and meta-analysis

**DOI:** 10.1186/s12879-019-4656-1

**Published:** 2019-12-04

**Authors:** Fasil Wagnew, Setegn Eshetie, Animut Alebel, Cheru Tesema, Getiye Dejenu Kibret, Alemu Gebrie, Getenet Dessie, Amanuel Alemu Abajobir

**Affiliations:** 1 College of Health Science, Debre Markos University, Debre Markos, Ethiopia; 2 College of Health Science, University of Gondar, Gondar, Ethiopia; 3 Faculity of health, University of Technology Sidney, Sidney, Australia; 4 College of Health Sciences, Bahirdar University, Bahirdar, Ethiopia; 5 Faculty of Medicine, The University of Queensland, Brisbane, Australia; 6 African Population and Health Research Center, Maternal and Child Wellbeing Unit, Nairobi, Kenya

**Keywords:** HAART, Children, Systematic review, Meta-analysis, Prevalence, Epidemiology, Human immunodeficiency virus, HIV, Ethiopia, Comorbidity, Iron status, Anemia

## Abstract

**Background:**

Anemia is a common problem in HIV (human immunodeficiency virus) infected patients, and is associated with decreased functional capacity and quality of life. Ethiopia is one of the countries which has expanded highly active antiretroviral treatment (HAART) over the past years. The effect of HAART on anemia among HIV remains inconsistent and inconclusive, particularly in children. This study thus aimed to synthesize the prevalence of anemia among HIV infected Ethiopian children and its association with HAART initiation.

**Methods:**

MEDLINE/PubMed, EMBASE, PsycINFO, Web of Science and Google scholar were used to identify 12 eligible studies reporting an association between anemia and HIV using a priori set criteria. PRISMA guideline was used to systematically review and meta-analysis these studies. Details of sample size, magnitude of effect sizes, including odds ratio (OR) and standard errors were extracted. Random-effects model was used to calculate the pooled estimates using STATA/SE version-14. I^2^ and meta-bias statistics assessed heterogeneity and publication bias of the included studies. Sub-group analyses, based on study designs, were also carried out.

**Results:**

In Ethiopia, the overall prevalence of anemia in HIV infected children was 22.3% (95% CI: 18.5–26.0%). The OR of anemia-HIV/AIDS comorbidity was 0.4 (95% CI, 0.2–0.5) in HAART initiated children as compared to non-initiated counterparts. Meta-bias and funnel plot detected no publication bias.

**Conclusion:**

On aggregate, anemia is a common comorbidity in pediatric HIV patients. HAART was significantly associated with a reduced anemia-HIV/AIDS comorbidity. Prompt start of HAART might help decreasing the prevalence of anemia and its subsequent complications.

## Background

Anemia has substantial negative effects on the health and economic wellbeing of nations and communities. Children with anemia experience irrevocable cognitive and developmental delays and exhibit decreased work related productivity as adults [1]. Anemia among HIV infected children is an emerging public health problem. A quarter of the global population suffer from anemia, including 293 million (47%) children younger than 5 years [2] whereby more than 100 million of these anemic children live in Africa [3]. Ethiopia is amongst the many countries which have expanded antiretroviral therapy (ART) coverage over the past years.

In Ethiopia, over 90% of new HIV infected children acquire the virus through vertical transmission. Of the estimated incidence of HIV infections, children account 13% of the estimated total HIV positive population in 2014 [4]. A systematic review and meta-analysis done in Ethiopia reports 9.93% pooled prevalence of mother-to-child-transmission (MTCT) of HIV [5]. Also, previous study conducted in southern Ethiopia shows a 4.2% HIV positive rate in infants, of whom 2.6% were born from mothers who were on preventive MTCT intervention [6]. This may indicate that the magnitude of MTCT has not significantly reduced, and the country’s plan to achieve zero MTCT has been facing numerous challenges. That said, the government of Ethiopia has scaled up its efforts to hastily decline and subsequently eliminate MTCT and apprehend the vision of HIV free, new generation by 2020. In addition, the Ethiopian Federal Ministry of Health (FMoH) has employed option B+ (test-and-treat) prevention of mother-to-child-transmission (PMTCT), an approach to avoid new HIV infection in newborns. According to option B+, all HIV infected women should be initiated on ART as soon as possible regardless of gestational age, CD4 count and clinical stage, and should be continued the treatment for the rest of their lives. Also, the preferred first line ART regimen for children is AZT or ABC + 3TC + EFV and the alternative first line ART regimen is AZT + 3TC + NVP or TDF + 3TC + EFV [7].

Hematological complications have been documented to be the second most common cause of morbidity and mortality among HIV positive children [8]. Indeed, anemia is a common and serious complication of both HIV infection and its treatment. That is, anemia is recognized as a major hematologic complication and has a significant impact on children’s survival, treatment outcomes and quality of life [9]. This involves a serum hemoglobin concentration of < 11 g/dl for pediatrics age group or that < 4.0 g/dl in very severe cases [10, 11].

In general, multi-factorial causes contribute to the increase in the burden of anemia among HIV patients. Some include opportunistic infections, change in immune system adaptability, nutritional deficiencies and side effects of HAART drugs including bone marrow suppression by HAART. Other common causes of anemia in HIV are chronic diseases, and hemolytic anemia induced by oxidant drugs. HIV infection itself can also cause anemia, perhaps as a consequence of HIV infection of stromal cells [12, 13]. Although HAART is capable of reducing the incidence of anemia [14–17] through enhancing CD4 cell count and suppressing viral duplication, it can also cause anemia. For instance, cotrimoxazole, pentamidine and zidovudine often argued to be associated with reticuloendothelial iron block [18, 19]. Cytokines such as interleukin 1, tumor necrosis factor and the interferon play a role in impairing erythropoietin response by reducing concentration of marrow progenitors and erythroid colonies. Zidovudine is widely used drug that results in myelosuppression causing anemia [12]. In the same vein, although HAART has been associated with a decrease in the incidence and severity of anemia, recent evidence shows that a decrease in hemoglobin level still occurs and is a predictors of poor clinical outcome.

Moreover, according to EDHS 2016 reports, 57% of children age 6–59 months suffered from some degree of anemia (hemoglobin levels below 11 g/dl). Twenty-five percent of children are classified with mild anemia, 29% with moderate anemia, and 3% with severe anemia [20]. Sadly, the trend of anemia prevalence among Ethiopian children declined from 54 to 44% from 2005 to 2011, but increased to 57% in 2016 [20]. This high prevalence of anemia in 2016 may be due to iron deficiency is a key factor of hemoglobin, and iron deficiency is valued to be responsible for this high prevalence. Other attributes of anemia include malaria, hookworm and helminthes, nutritional deficiencies and chronic infections. In other words, although great advances have been made in the management of childhood HIV/AIDS, its burden continues to hamper progress in reducing childhood mortality and morbidity in much of the developing world, particularly in this region [21].

Nonetheless, there is limited evidence on the prevalence of anemia among HIV infected children in Ethiopia. Similarly, the evidence base on the prevalence and effect of HAART on children with HIV/AIDS and anemia comorbidity still remains inconsistence, uncertain and inconclusive. This may perhaps be due to paucity of data on the topic, particularly in HIV infected children. This systematic review and meta-analysis was therefore aimed to summarize the contemporary evidence base on the prevalence of anemia among children with HIV/AIDS and its association with HAART in Ethiopia.

## Methods

### Study design and search strategy

#### Study design

This is a systematic review and meta-analysis of published articles on anemia among HIV infected children below 18 years in Ethiopia.

To identify relevant studies, two authors (FW, SE) exhaustively and systematically searched for articles published in English from 1990 to 2017 in MEDLINE/PubMed, EMBASE, PsycINFO, Web of Science and Google scholar. Grey literature and reference lists such as programme reports were also retrieved. We used medical subject headings (MESH), adding terms and keywords from a primary search to formulate search strategy in these databases. In all databases, we consumed an interactive process to improve the search strategy through checking numerous search terms and including new search terms as new relevant citations were identified. The keywords included: as “HAART, children, systematic review, meta-analysis, prevalence, epidemiology, human immunodeficiency virus, HIV, Ethiopia, comorbidity, iron status, anemia”. Boolean operators – ‘OR’ or ‘AND’ – were used*.* Endnote reference manager software was utilized to collect and organize search outcomes and for removal of duplicate articles. Moreover, we followed the Preferred Reporting of Systematic Reviews and Meta-Analysis (PRISMA) guideline [22]. Furthermore, the study period for searching of articles were carried out from the beginning of August, 2017 until December, 2017.

### Inclusion criteria

The inclusion criteria included both cross-sectional and cohort studies with baseline measures for the outcome of interest (anemia in HIV) using a priori *criteria*. In this context, anemia was defined based on the WHO cutoff point (hemoglobin less than 11 g/dl) for children below 18 years.

### Exclusion criteria

Case reports, case reviews and studies addressing specific groups such as children with other hemoglobinopathies were excluded.

### Data extraction

Two reviewers (FW and SE) screened the downloaded titles and abstracts using the eligibility criteria. Discussions and mutual consensus were in place when possible arguments were raised between the two reviewers. The two reviewers then assessed the full text of potentially eligible papers. Whenever further information is required, we made some efforts to contact the author’s by email. When OR with 95% Confidence Interval (CI) was not computed in the result, we used numerator and denominator data and beta coefficients and their standard errors. The following study characteristics were extracted: primary author, year of publication, study area, study design and sample size. The proportion of anemia was also retrieved from each included study.

### Quality appraisal

Articles were assessed for quality, with only high quality studies included in the analysis. Two reviewers (FW, SE) independently extracted the necessary information from the relevant articles. Discrepancies were adjudicated or discussed with coauthors (GD, AAA), whenever appropriate. Newcastle-Ottawa Scale modified for prevalence studies methodological quality assessment tool was utilized [23]. For dichotomous data, we extracted the number of participants with the outcome and the total number sample size. FW and AAA were involved in conception of the research questions and design as well as critical review of the paper.

### Data analysis

Relevant information about the study area, study design and study sample were summarized by Microsoft Excel and then exported to STATA/SE version-14 for further analysis. The pooled prevalence of anemia was conducted using a random-effects model with 95% CI. Heterogeneity among studies was computed using the I^2^ statistic [24]. The I^2^ statistic estimates the percentage of total variation across studies. In this study, forest plots were also utilized to estimate pooled effect size and weight of each recruited study with 95% CI to show a graphic summary of the data. For meta-analyses with a minimum of 10 studies, publication bias was determined based on the visual assessment of the funnel plot [25] and Egger’s test [26]. Sub-group analysis by study design was employed to resolve the occurrence of high heterogeneity in the included studies. In an effort to understand the sources of heterogeneity meta-regressions were performed on sample size and year of publication. Meta-regression was used instead of subgroup analyses as it allows for the use of continuous covariates and permits the inclusion of more than one covariate at a time.

## Results

**Fig. 1:**
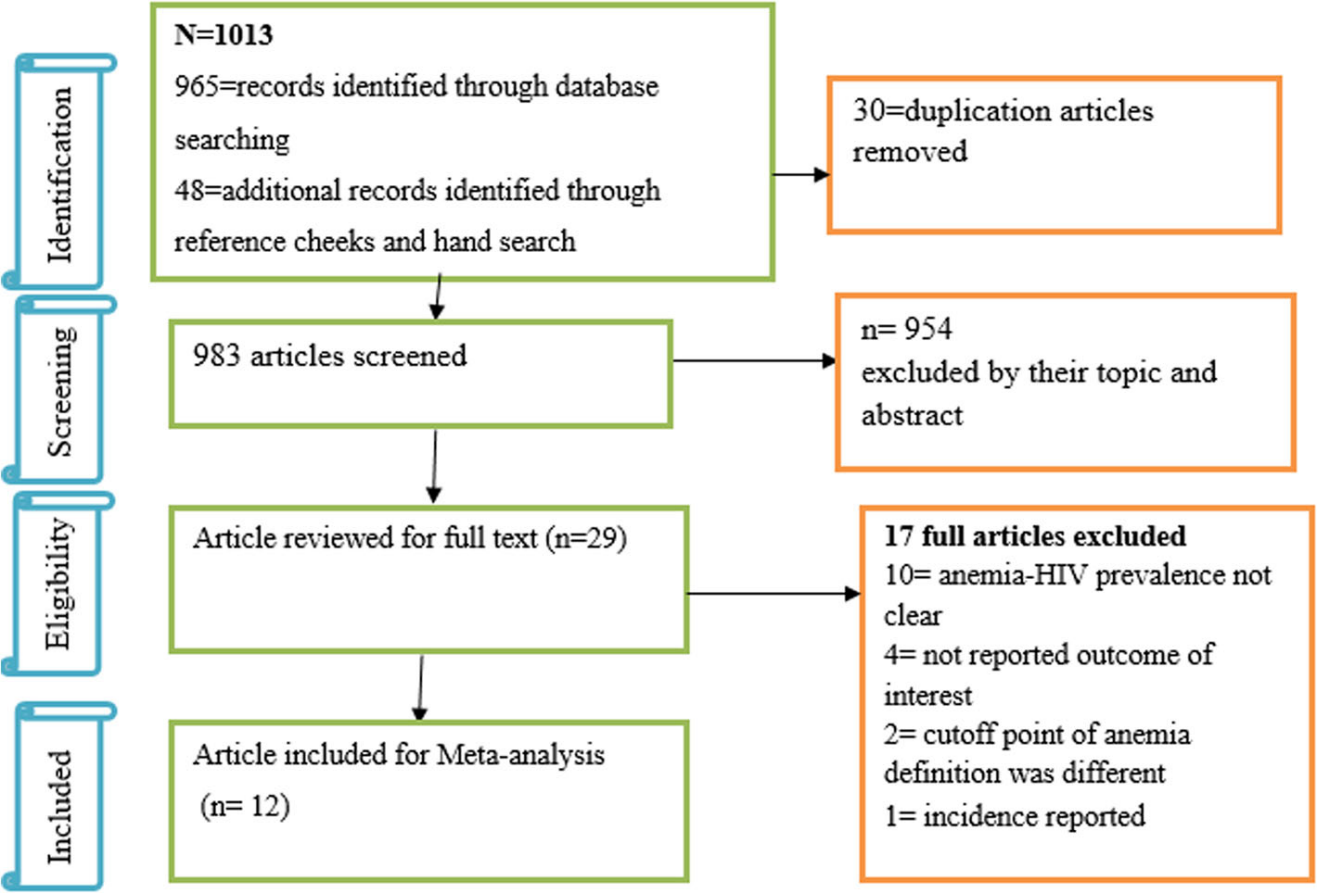
Flow chart describing selection of studies for a systematic review and meta-analysis of HIV-anemia comorbidity among children in Ethiopia

We followed the PRISMA guideline to present the findings of this review. First, 1013 articles related to the prevalence of anemia in HIV-positive children were found. Of these, 30 duplication and 954 unrelated articles were excluded. Second, from the remaining 29 articles, 17 full-text articles were excluded. Among these, 10 studies [27–36] did not present relevant findings exclusively for study target population and, 7 studies were excluded because 1 was incidence study [37], 4 studies [38–41] did not present outcome of interest and 2 studies [42, 43] used various hemoglobin cutoff points. Finally, 12 articles representing 3524 children living with HIV, met the inclusion criteria (Fig. 1).

### Descriptive summary of the included full-text articles

All the 12 studies representing 3524 children included in the present review provided data on prevalence of anemia among HIV infected children. The mean age of participants was 7.7 years with standard deviation (SD) of +3.3 Years. The prevalence of anemia among individual studies ranged between 12.3% (in Amhara region) [25] to 39.4% (in Harer) [17] (Table 1)

**Table 1:** Descriptive summary of 12 included studies on the anemia-HIV- comorbidity in children on HAART in Ethiopia

Authors and year	Region of study	Types of hospital	Study design	Sample size	Number of children with outcome	Prevalence (%)	Study quality score
Muluneh A. et al.,2009 [18]	Oromia	Referral	Cross-sectional	64	14	21.87	8
Koye et al.,2012 [44]	Amhara	Referral	cohort	520	103	19.80	7
Kedir et al., 2014 [45]	Oromia	Referral	cohort	522	109	20.88	6
Enawgaw et al.,2015 [46]	Amhara	Referral	Cross-sectional	265	43	16.22	8
Teklemariam et al.,2015 [17]	Harer	Referral	Cross-sectional	66	26	39.39	6
Hylemariam et al.,2015 [16]	Addis Ababa	Referral	Cross-sectional	180	40	22.22	7
Debasu et al.,2015 [47]	Addis Ababa	Referral	Cross-sectional	106	20	18.86	7
Tesfanesh.A,2016 [48]	Adis Abeba	Referral	Cross-sectional	108	19	17.59	8
Tsegay et al.,2017 [49]	Amhara	Referral	Cross-sectional	224	66	29.46	8
Animut et al.,2017 [50]	Amhara	Referral	cohort	538	66	12.26	8
Bitew et al.,2017 [51]	Wolaita	HC	cohort	228	77	33.77	9
Lamessa D.,2017 [52]	Addis Ababa	hospital	cohort	703	168	23.89	7

### Descriptive summary of HAART on anemia-HIV/AIDS comorbidity

Four studies were included to determine the effect of HAART on anemia among HIV/AIDS children. These studies reported OR ranging from 0.2 [16, 18] to 0.5 for the comorbidity [17] (Table 2).

**Table 2:** The effect of HAART treatment on HIV/AIDS-anemia comorbidity

Region	Author and year		Anemic	Non-anemic	OR
Oromo	Muluneh A. et al.,2009 [18]	HAART	14	50	0.24
		Non-HAART	34	30	
Amhara	Enawgaw et al.,2015 [46]	HAART	24	164	0.447
		Non-HAART	19	58	
Harer	Teklemariam et al.,2015 [17]	HAART	20	46	0.49
		Non-HAART	31	35	
A.Ababa	Hylemariam M.,2015 [16]	HAART	8	71	0.24
		Non-HAART	32	69	

### Comorbidity of anemia-HIV/AIDS in children

**Fig. 2:**
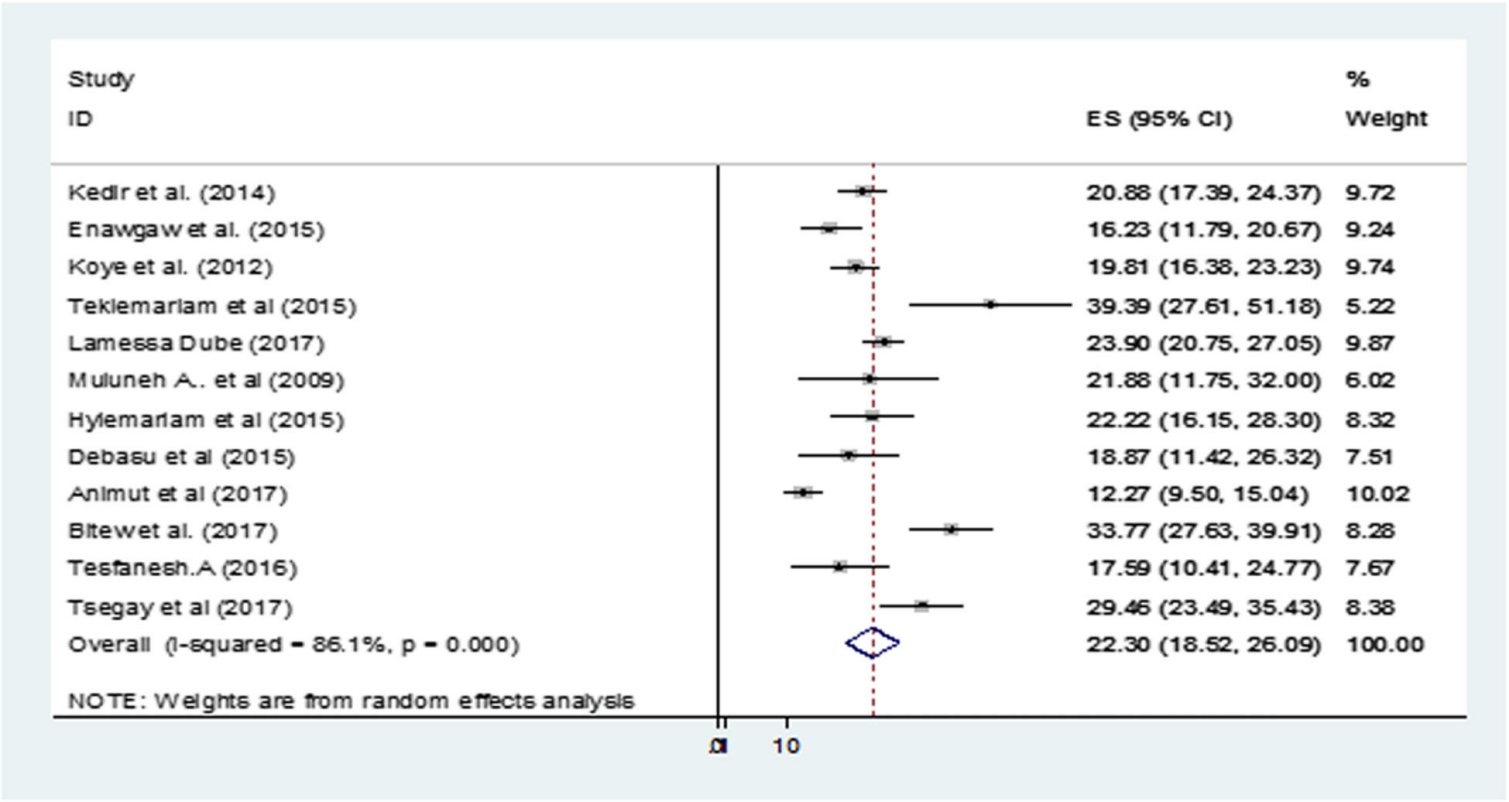
Forest plot showing the point prevalence of anemia-HIV comorbidity in children in Ethiopia

**Fig. 3:**
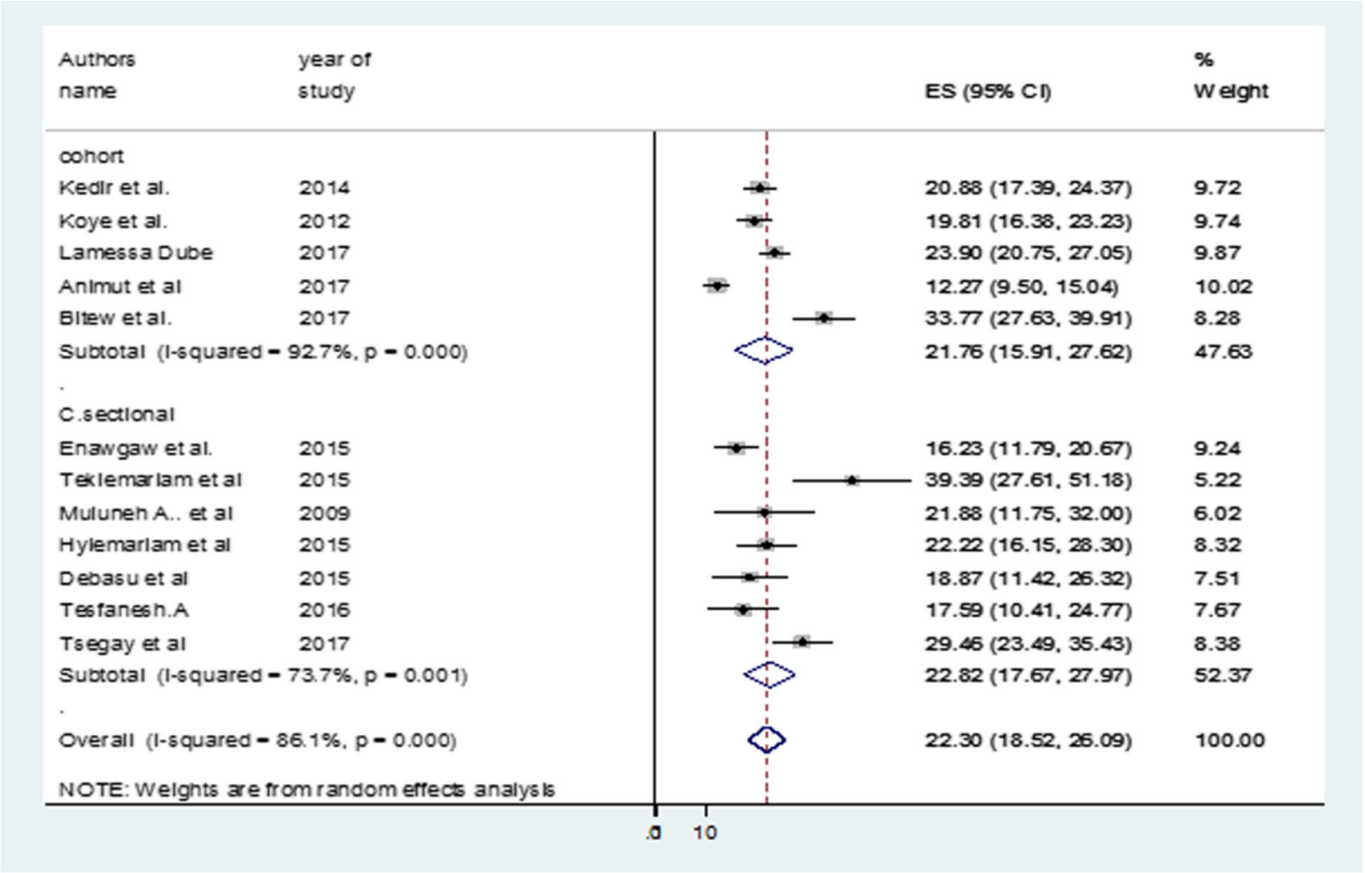
Forest plot showing subgroup analysis of anemia-HIV comorbidity in children in Ethiopia by study designs

The point prevalence of anemia among HIV/AIDS infected Ethiopian children was 22.3% (95% CI: 18.5–26.1) (Fig. 2). A sub-group analysis by study designs also showed similar levels of comorbidity in these children (Fig. 3). The I^2^ test indicated high heterogeneity (I^2^ = 97.7%, *p* < 0.001). In an effort to identify the possible source of heterogeneity, different factors associated with the heterogeneity such as publication year and sample size of the study. These were investigated by using univariate meta-regression models, but none of these variables were statistically significant (Table 3).

**Table 3:** Related factors with heterogeneity of the anemia-HIV comorbidity in the current meta-analysis (Based on Univariate Meta Regression)

Variables	Coefficient	*P*-value
Publication year	.6539374	0.53
Sample size	−.0112698	0.3

### The pooled effect of HAART on anemia in HIV/AIDS infected children

**Fig. 4:**
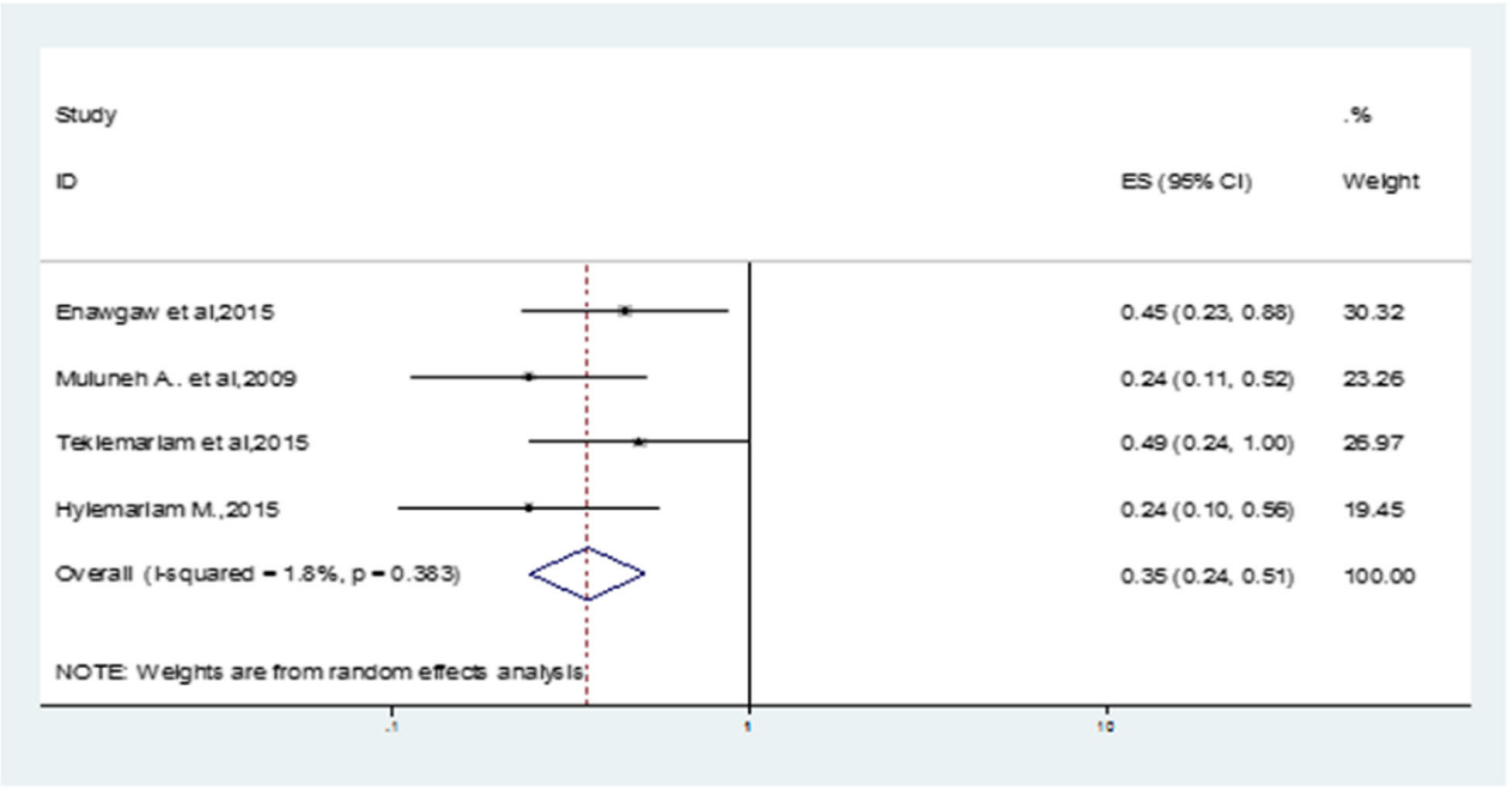
Forest plot showing the effect of HAART treatment on anemia-HIV comorbidity in children in Ethiopia

Treatment with HAART for children living with HIV/AIDS was found to decrease the level of anemia by 60% (OR: 0.4 (95% CI (0.2–0.5)) as compared to non-HAART initiated children with the disease. Heterogeneity test showed no statistically significant evidence, *p* = 0.38 (Fig. 4). Also, the overall Egger’s test for publication bias revealed no statistically significant evidence, *p*-value = 0.17 (Additional file 1: Figure S1).

## Discussion

The evidence on the pediatric HIV infection and anemia is limited because of paucity of data on anemia associated with HIV infection as well as on the effect of HAART on the anemia. Using the WHO definition of pediatric anemia (hemoglobin < 11 g/dl), more than 1 in 5 children living with HIV/AIDS are anemic in Ethiopia. This finding is in keeping with the global perspective of the burden of the disease in that mild and moderate anemia is more prevalent with HIV infection irrespective of region [53]. Interestingly, however, this finding is slightly lower than findings from previous studies. For example, a systematic review and meta-analysis done by Esan et al. [54] suggested a 34% pooled mean prevalence of anemia in HIV-infected children. Furthermore, a study of 2919 HIV-positive children from west Africa shows 40.5% rate of anemia in children [55]. The observed difference might be attributable to the differences in sociodemographic characteristics, study design and study period. Iron, B12 and folic acid deficiencies, local nutritional status and prevalence of parasitic infections such as malaria and/or hookworm might contribute to the variations in the prevalence of anemia. Moreover, a possible reason for the decreased prevalence of anemia in this study could be attributed to the adoption of the recent “WHO 90-90-90 test and treat policy” which has seen many more HIV infected children initiated early on HAART thereby halting anemia comorbidity [56, 57]. Although investigation into the mechanisms associated with anemia in HIV patient is beyond the scope of the current study, it could also be explained by a decreased production, increased destruction and ineffective production of red blood cells. Furthermore, abnormal nutrient absorption and diseases of the gastro-intestinal system, particularly in advanced HIV/AIDS cases, may lead to anemia [58].

This study also showed that HAART significantly reduced anemia in children living with HIV/AIDS. That is, children on HAART are less likely to be anemic as compared to their anemia status before treatment with HAART is initiated or relative to non-HAART initiated children. This finding is in line with study done by Walker et al. (2002) that revealed that the occurrence of anemia-HIV/AIDS comorbidity was lower in children receiving routine HAART as compared to children not on HAART [59]. This evidence is also supported by other previous studies [54, 60–62] that revealed the prevalence of anemia was higher in HIV infected children who did not receive HAART. The capacity of HAART to prevent opportunistic infections could in some way reduce the negative impact of retroviral infection on bone-marrow cells. Moreover, HAART-enhanced hematopoietic progenitor cell growth may reduce viral load by inhibiting viral replication [63, 64]. Overall, the findings of this study suggested the importance of HAART to mitigate the clinical and public health consequences of anemia among pediatric HIV/AIDS patients.

However, the findings need to be considered in the context of some important limitations. Even though we performed subgroup analyses and meta-regression, heterogeneity was observed in all analyses. These include the inclusion of studies published only in English that may compromise representativeness (due to language bias). As well, because of heterogeneity across studies and lack of sufficient number of studies, this study did not explore other potential factors contributing to anemia in HIV/AIDS such as the type of HAART, the length of antiretroviral exposure, presence of opportunistic infections and advanced stage of the HIV/AIDS disease. Future research that considers these variables and disease characteristics would advance the findings of this meta-analysis.

## Conclusion

Anemia is a common comorbidity in children living with HIV/AIDS in Ethiopia. HAART initiation was significantly associated with a reduced anemia-HIV/AIDS comorbidity. Prompt start of HAART, whenever appropriate, is suggested to decrease the prevalence of the comorbidity and subsequent complications.

## Supplementary information

**Additional file 1: Figure S1.** The overall Egger’s test for publication bias revealed the included studies.

## Data Availability

All data generated or analysed during this study are included in this published article [and its supplementary information files].

## Abbreviations

HAART: Highly Active Antiretroviral Treatment
HIV/AIDS: Human Immunodeficiency Virus/Acquired Human-Immune Deficiency Virus
MTCT: Mother to Child Transmission
OR: Odds Ratio
WHO: World Health Organization
