# Supplementary figures and images for: Burden of anemia and its association with HAART in HIV infected children in Ethiopia: a systematic review and meta-analysis
Source: BMC Infect Dis. 2019 Dec 4;19:1032. doi: 10.1186/s12879-019-4656-1 (PMC6894299; doi:10.1186/s12879-019-4656-1)

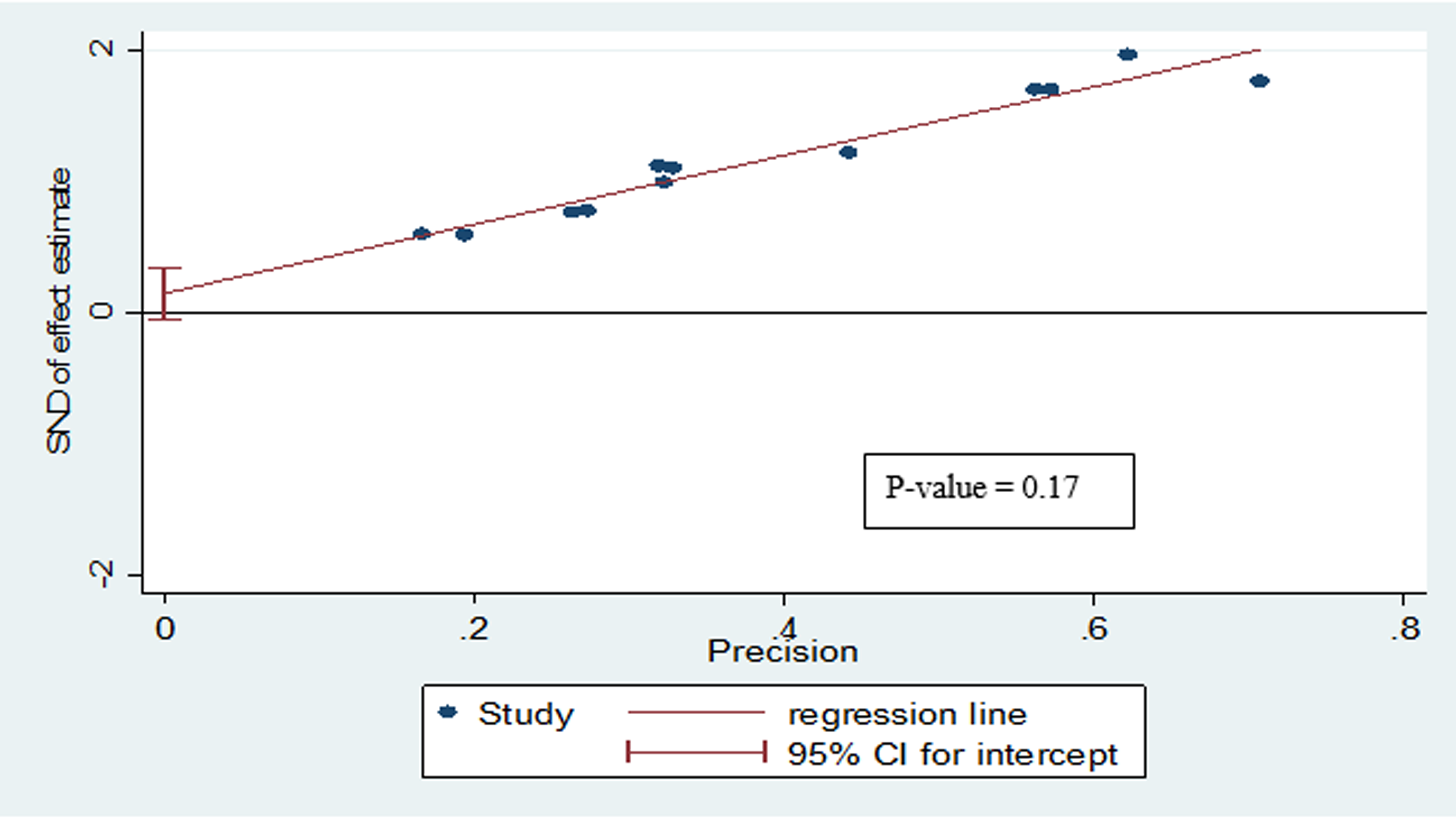

Supplement: Supplementary file 1 — Additional file 1: Figure S1. The overall Egger’s test for publication bias revealed the included studies. [file 12879_2019_4656_MOESM1_ESM.png]
